# Forty Years Since the Structural Elucidation of Platelet-Activating Factor (PAF): Historical, Current, and Future Research Perspectives

**DOI:** 10.3390/molecules24234414

**Published:** 2019-12-03

**Authors:** Ronan Lordan, Alexandros Tsoupras, Ioannis Zabetakis, Constantinos A. Demopoulos

**Affiliations:** 1 Department of Biological Sciences, University of Limerick, V94 T9PX Limerick, Ireland; Alexandros.Tsoupras@ul.ie (A.T.); Ioannis.Zabetakis@ul.ie (I.Z.); 2 Health Research Institute (HRI), University of Limerick, V94 T9PX Limerick, Ireland; 3 Department of Chemistry, National and Kapodistrian University of Athens, Panepistimioupolis, 15771 Athens, Greece; Demopoulos@chem.uoa.gr

**Keywords:** platelet-activating factor, inflammation, cardiovascular disease, cell signalling, phospholipids

## Abstract

In the late 1960s, Barbaro and Zvaifler described a substance that caused antigen induced histamine release from rabbit platelets producing antibodies in passive cutaneous anaphylaxis. Henson described a ‘soluble factor’ released from leukocytes that induced vasoactive amine release in platelets. Later observations by Siraganuan and Osler observed the existence of a diluted substance that had the capacity to cause platelet activation. In 1972, the term platelet-activating factor (PAF) was coined by Benveniste, Henson, and Cochrane. The structure of PAF was later elucidated by Demopoulos, Pinckard, and Hanahan in 1979. These studies introduced the research world to PAF, which is now recognised as a potent phospholipid mediator. Since its introduction to the literature, research on PAF has grown due to interest in its vital cell signalling functions and more sinisterly its role as a pro-inflammatory molecule in several chronic diseases including cardiovascular disease and cancer. As it is forty years since the structural elucidation of PAF, the aim of this review is to provide a historical account of the discovery of PAF and to provide a general overview of current and future perspectives on PAF research in physiology and pathophysiology.

## 1. Platelet-Activating Factor

Since its discovery, the structure of platelet-activating factor (PAF) also known as PAF-acether or AGEPC (acetyl-glyceryl-ether-phosphorylcholine) has been identified as a phosphoglycerylether lipid mediator involved in diverse physiological and pathophysiological processes. It seems apparent that PAF has different physiological roles in animals, plants, and monocellular organisms. It is considered the most potent lipid mediator known to date [1,2]. Previous to the 1970s, lipid mediators were thought to be generally derived from phospholipids. However, PAF was the first intact phospholipid mediator to demonstrate autacoid or messenger functions [3]. PAF was initially considered one molecule, which is commonly referred to as the classical PAF. Now it is understood that there are a large number of structurally related phospholipids or PAF analogues that are dissimilar in structure to PAF that interact with the PAF-receptor (PAF-R) and belong to the ‘PAF family’, collectively known as PAF-like lipids (PAFLL). However, for the purpose of this review, PAF refers to the classical structure reported in 1979, which is responsible for most of the known biological effects and is thought to be the most potent PAF molecule. PAF mediates a wide variety of cellular functions and cell–cell interactions. Therefore, PAF is involved in several physiological processes including apoptosis, physiological inflammation, wound healing, reproduction, angiogenesis, long-term potentiation, and potentially retrograde signalling [4,5,6,7]. However, PAF is also a potent pro-inflammatory mediator that is implicated in a variety of conditions and chronic diseases such as cancer, renal diseases, cerebrovascular and central nervous system disorders, allergies, asthma, infections, and cardiovascular diseases (CVD) [5,8,9,10,11,12,13]. PAF is known to carry out its broad pathophysiological actions at concentrations as low as 10^−12^ M and almost always by 10^−9^M as an intercellular messenger [14]. In evolutionary terms, many ether lipids were replaced over time by their esterified analogues; however, PAF and other minor phosphoglycerylether molecules were conserved in various organisms due to their important biological roles [15]. Hence, the importance of understanding the role of PAF in various biological processes. The aim of this review is to provide a historical account on the discovery of PAF, the research conducted since, and to provide future research perspectives on PAF research in general.

## 2. The Discovery and Structural Elucidation of the Platelet-Activating Factor

### 2.1. The Discovery of the Platelet-Activating Factor

PAF was first introduced into the literature in 1966 when Barbaro and Zvaifler described a substance that caused antigen induced histamine release from rabbit platelets producing antibodies in passive cutaneous anaphylaxis [16]. Almost four years later, Henson described a ‘soluble factor’ released from leukocytes that induced vasoactive amine release in platelets. Further observations by Siraganuan and Osler [17] described the existence of a diluted substance that had the capacity to cause platelet activation. A year later Jacques Benveniste and colleagues elaborated on the findings of the previous two studies and described a novel factor that induced aggregation and secretion of platelets, which participated in a leukocyte-dependent histamine release from rabbit platelets [18]. Hence, the term platelet-activating factor (PAF) was coined because of the initial observations of its effects on platelets [18]. It was later discerned that PAF was a lipid-like molecule [19]. It is recalled that to study PAF Benveniste prepared a measure of PAF from 100 L of hog blood, which resulted in a 100 L solution from which 1 µL was sufficient to induce platelet aggregation, indicating its high level of potency [20]. However, this amount of PAF was too low to use techniques at the time such as mass spectrometry or magnetic resonance that might determine the structure of the bioactive compound [20]. Despite the lack of structural data, Benveniste and others had determined several of the physical characteristics of PAF. They determined that it was a lipid compound, it could bind to albumin, and it migrated between lysolecithin and sphingomyelin in thin-layer chromatography separation, all properties of which were similar to that of lysophosphatidylcholine. The compound was also affected by several phospholipases (PLA_2_, PLC, and PLD) but resistant to others (sphingomyelinase C and PLA_1_), indicating that indeed it had a phospholipid type structure [20,21]. Studies began to discern that PAF was implicated in IgE anaphylaxis [22] and many of the properties of PAF released during IgE anaphylaxis began to be elucidated [23]. Furthermore, the role of PAF in platelet aggregation was beginning to be further understood by June 1979 [24].

### 2.2. Structural Elucidation of the Platelet-Activating Factor

**Figure 1:**
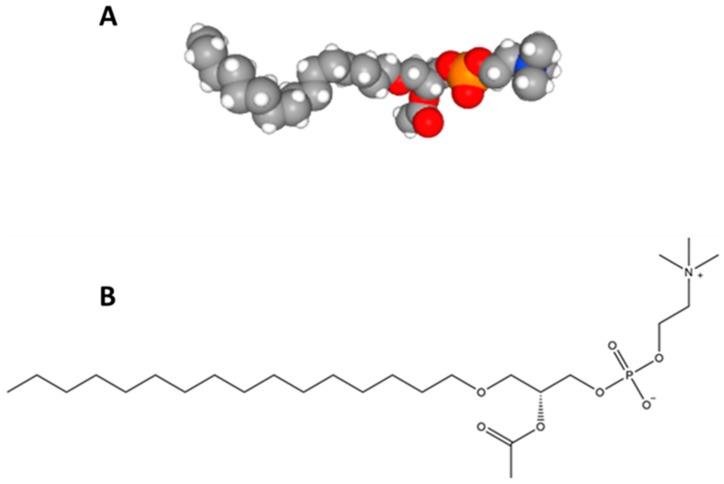
The structure of platelet-activating factor (PAF): (**A**) PAF space fill model data from [42] and (**B**) PAF structural model.

Following several experiments with phospholipases, etc. the structure of PAF was thought to be 2-acyl-*sn*-glycero-3-phosphocholine (1-lysophosphatidylcholine) [25], but owing to acyl chain migration this molecule was known for its instability and did not demonstrate the biological properties corresponding to PAF [20,26]. Around that time, several other structures were interrogated, and many researchers were involved in discussions as reviewed by Chap [20]. However, on the 10th of October 1979, Constantinos Demopoulos, Neal Pinckard, and Donald Hanahan, from San Antonio Texas published the structure of PAF (1-*O*-alkyl-2-acetyl-*sn*-glycero-3-phosphocholine) under the name AcGEPC (Acetyl-glyceryl-ether-phosphocholine), which was shown to have biological activities indistinguishable from that of naturally generated rabbit PAF (Figure 1) [27]. The researchers realised that the AcGEPC they synthesised was indeed the same structure as naturally occurring PAF. Interestingly, nineteen days after the Demopoulos, Pinckard, and Hanahan [27] publication, the same structure was reported by a group led by Fred Snyder who were assessing the properties of an isolated compound in the kidney that was responsible for peculiar biological activity, which was known by them as the antihypertensive polar renomedullary lipid (APRL) [28]. These studies were followed by an article by Benveniste who subsequently proposed the name PAF-acether [29]. Later articles confirmed that synthetically produced PAF initiated identical biological effects to the PAF molecules responsible for IgE-induced systemic anaphylaxis [30], which also caused similar vascular, cardiovascular, and respiratory problems associated with anaphylaxis in rabbits [31] and baboons [32]. In addition, platelets were not required to induce anaphylactic shock in rabbits when injected with synthetic PAF, indicating for the first time that PAF acts via a receptor [33].

Hanahan and colleagues formally confirmed the structure of PAF in 1980 using mass spectrometry and simplified their abbreviation of the molecules name to AGEPC [34]. Likewise, Benveniste and colleagues simplified the name of the PAF precursor to lyso-PAF [35]. As many researchers were working with PAF at the same time, it is reported that there were conflicting attitudes between the groups with reference to what the name of the molecule should be. Furthermore, Chap described the difficulty encountered by Benveniste who was unfortunate not to have elucidated the structure of PAF previous to the other groups [20]. Considering that we now know PAF exhibits a vast diversity of actions and the fact that a myriad of other molecules can activate platelets, it seems ironic that the name PAF is a misnomer [36] that has remained in the literature.

However, that was not the end of Benveniste’s role in determining some of the properties of PAF. Indeed, Benveniste and colleagues provided the first evidence that platelets synthesise PAF [37] and they determined the subcellular localisation of PAF biosynthesis in human neutrophils [38]. However, Benveniste’s important role in the discovery of PAF may be overshadowed by his later controversial research that led to major scientific scandals [20,39,40] that are not the subject of this review. The very first account of the discovery of PAF and its various properties was published in Nature in 1980 by Cusack [41]. The intensive and dedicated research of many scientists involved in the discovery and structural elucidation of PAF in the 70s and 80s set in motion a research field that is ever growing to this day, which has had profound implications to medical research.

## 3. The Importance of Platelet-Activating Factor Research

PAF is implicated in various physiological processes and a multitude of pathophysiological processes, as will be further discussed in Section 3.1 and Section 3.2. However, the critical feature of PAF physiologically and in disease is that the biological effects of PAF can be modulated by diet, lifestyle, and environmental factors [5,43,44,45,46]. This means that PAF could be a potential therapeutic target for many chronic diseases [5,8,10] and thus PAF is of significant importance and value to researchers across several disciplines. Section 3.1 and Section 3.2 discuss some of the main biological consequences of PAF signalling in physiology and pathophysiology. While this review discusses many of these events, not all of PAF’s roles are discussed due to the vast accumulation of research published around PAF in the last forty years. In the last two years alone there has been over 2000 articles published in relation to PAF. This review specifically focuses on some of the emerging PAF-related research trends over the last decade. In particular, this article discusses the most contentious issues of PAF research such as the role of the PAF metabolic enzymes in physiological and inflammatory processes and the role of PAF in various chronic diseases, such as disorders of the central nervous system (CNS), CVD, and cancer. These diseases have major health implications for patients and are an enormous burden to healthcare globally. Indeed, some of the research highlighted in this article may lead to ground-breaking discoveries that enhance our understanding of cell signalling, inflammation, and disease.

After the elucidation of the structure of PAF in 1979, there was much motivation in the development of research in the field that from 1983 lead to several congresses being organised entirely focused on PAF research. These congresses were held every three years worldwide until 2004 (Table 1). After 21 years, PAF research became interdisciplinary and grew and expanded to virtually all areas of biochemistry and medicine. The congresses stopped being organised as much of the research surrounding PAF were disseminated at various international conferences. However, attempts have been made to reignite these congresses as recent as February 2015 in Tokyo Japan, where PAF communications were presented in special sessions at the ‘6th International Conference on Phospholipase A_2_ and Lipid Mediators’ [47].

### 3.1. PAF Signalling in Physiology

PAF was initially thought to be a single molecule, but as aforementioned, it became clear that there were other structurally related phospholipids or PAFLL [14]. These molecules have semi-similar or non-similar structures and exhibit similar biological activities to PAF [48]. PAF and PAFLL are structurally defined ligands of the PAF-R, which has restricted expression on specific target cells of the immune, haemostatic, and inflammatory systems [12]. PAF itself is synthesised constitutively or under appropriate stimuli by a variety of cells such as platelets, macrophages, monocytes, neutrophils, basophils, eosinophils, mast cells, and endothelial cells [49]. PAF is synthesised by two markedly different pathways known as the de novo and remodelling pathways. Fred Snyder aforementioned was instrumental to the discovery of the anabolic and catabolic enzymes of PAF metabolism [50]. His research group was responsible for discovering the roles of lyso-PAF acetyltransferase (Lyso-PAF-AT) as the main regulatory enzyme of the remodelling pathway [51], PAF acetylhydrolase as the main regulatory enzyme of PAF catabolism [52], a novel DTT (dithiothreitol)-insensitive CDP-choline phosphotransferase as the main regulatory enzyme of the de novo pathway [53], and other enzymes implicated in PAF metabolism [50]. It was thought that the remodelling enzymatic pathway of PAF biosynthesis was responsible for the pro-inflammatory production of PAF in acute and chronic inflammation.

**Figure 2:**
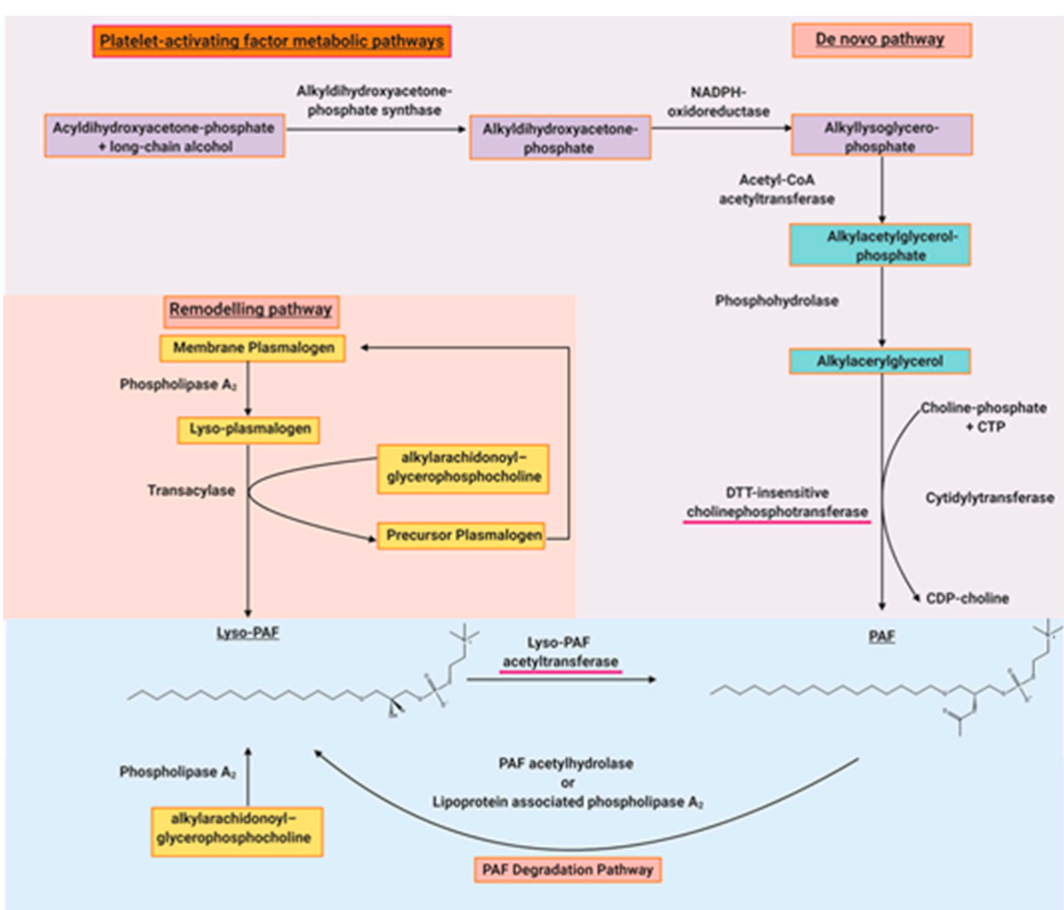
The main biosynthetic and degradation pathways of PAF. The red underlined enzymes are the regulatory enzymes of PAF synthesis.

On the other hand, the de novo pathway was initially thought to be responsible for the constitutive production of PAF, maintaining basal PAF levels. As a result, the de novo pathway was neglected with regard to research in inflammation. However, it is now recognised that PAF-CPT, a key enzyme of the de novo pathway, seems to be more active during chronic inflammatory manifestations [54]. Consequently, there is an increase in the basal levels of PAF related to the continuous activation of inflammatory cascades during the development of inflammation-related chronic disorders [54,55,56,57]; hence the regulation of the biosynthetic pathways of PAF seem to be more complicated than previously envisaged. PAF biosynthesis is correlated with well-established inflammatory and immunological biomarkers of inflammation in various chronic disorders [8,56,57,58,59,60]. Nevertheless, the role of these enzymes and others in PAF metabolism and disease is the subject of intensive research by a few research groups, which have previously been reviewed [5,61,62,63,64,65,66]. There is considerable debate over PAF-acetylhydrolase (PAF-AH), which is a catabolic enzyme of PAF; however, it may play a role in in the development of atherosclerosis [65], which is elaborated on in Section 5.3. PAF-AH inactivates PAF by removing the acetyl-group at the *sn*-2 position, which is a key feature of the regulation of circulating PAF levels [8]. The actions and roles of the various metabolic enzymes of PAF are summarised in Figure 2.

The signalling functions of PAF are mostly associated with acute and chronic inflammation in essentially all organs, which are well characterised in the literature [3,12]. However, an acute inflammatory response can be considered both a physiological and pathophysiological function of PAF as it is necessary for the day-to-day protection of tissue from pathogenic insults. PAF can mediate events in a juxtacrine, paracrine, autocrine, and endocrine manner. In acute inflammation, PAF is synthesised by endothelial cells stimulated with thrombin or other inflammatory mediators. This PAF then activates polymorphonuclear leukocytes (PMNs), which are the first leukocyte responders and key effectors cells of the acute inflammatory response to accumulate at an inflamed site [3,67,68]. These events were the first evidence of synthesis of a signalling factor for PMNs by inflamed endothelial cells mediated by activation-dependent alterations in the affinity and avidity of β_2_ integrins on the surface, which established that there was a molecular mechanism for the activation leukocytes at the endothelial cell surface, rather than relying on the diffusion of chemotactic factors into the blood [3,69]. This led to further research demonstrating that PAF can induce a myriad of effects in PMNs and other leukocytes, such as monocytes, whereby PAF is involved in NF-κB translocation and alterations of gene expression [70]. Many of these signalling cascades and complex interactions are reviewed by Prescott, Zimmerman, Stafforini, and McIntyre [3].

Apart from acute inflammation, PAF is involved in cell signalling mechanisms for a number of other physiological processes. For instance, PAF has several surprising roles in reproduction physiology. Indeed, PAF signalling modulates female reproductive events including ovulation, fertilisation, preimplantation, implantation, and parturition. It is also thought that PAF plays a role in male reproduction due to the presence of PAF in spermatozoa, which may be involved in the induction of acrosome reaction and sperm motility [71,72,73,74,75,76].

Phospholipids predominate in the brain and play several critical structural and physiological roles [77]. Therefore, it is unsurprising that PAF also seems to play a crucial role in cell signalling of the CNS. PAF is synthesised by neural cells spontaneously or following appropriate stimuli [78,79] and the presence of the PAF-R in brain membranes has been reported since 1988 [80]. Neuronal PAF actions are associated with signal signalling processes mediated by phospholipase C (PLC) and protein kinase C (PKC) [81]. PAF activity in the brain is not limited to its proinflammatory function, neurotoxicity, and apoptosis [82], but it is also associated with neurotrophic effects [78]. Most notably, the PAF-R is present on intracellular membranes and in the synaptic membranes of the cerebral cortex. Indeed, it has been established that there is a relationship between PAF and the glutamate receptor in the CNS [4,83,84]. Therefore, it is clear that PAF is a critical mediator in CNS physiology. However, PAF may also be involved in CNS pathology, which warrants further research.

PAF is also a mediator of regular cardiovascular-related physiology as it involved in the mediation of blood pressure and normal inflammatory and haemostatic responses [85,86,87]. Hence why the term ‘platelet-activating factor’ was coined. However, PAF is mostly known for its role in inflammatory cascades that lead to the development of chronic diseases such as CVD and cancer, which are discussed in Section 3.2.

### 3.2. PAF Signalling in Pathophysiology

It is well-known that PAF is involved in a wide range of inflammation-related conditions and diseases [85]. As CVD is still the leading cause of global mortality [88], determining the causes and how to prevent CVD is imperative and a major objective of modern medicine [45]. Systemic inflammation mediates all stages of atherosclerosis [89] in which PAF is a key inflammatory mediator [5,10,11]. Several inflammatory mediators facilitate an interplay and crosstalk between various cells and endothelial cells, which initiate inflammatory cascades that eventually results in endothelial dysfunction and initiation of proatherogenic events [5,90]. PAF is one of the critical junctions between several inflammatory pathways (cytokines, oxidative stress, eicosanoids, etc.) that modulated the interplay between various cells participating in atherosclerosis [5]. PAF is synthesised by many of the key cells (when activated) involved in the atherosclerotic process, including endothelial cells, macrophages, monocytes, platelets, and even foam cells. Consequently, PAF is implicated in the initiation of atherosclerosis and continues to be involved through plaque formation, development, erosion, and rupture [5,10,11,91,92]. PAF levels can be increased by upstream mediators (IL-1β, IL-6, TNF-α, PAF itself, etc.) and in itself induce the production of downstream mediators [5]. In 2003, Demopoulos et al. [10] proposed that PAF is the molecular link between several theories of atherosclerosis development due to the fact that many of the atherogenic properties of oxidised low-density lipoproteins (LDL) can be attributed to the activity of PAF and PAFLL. PAF was termed ‘the missing link’ that orchestrates thrombosis, inflammation, and oxidation responsible for atherogenesis.

As a result of decreased PAF-AH activity and a deficiency of endogenous or ingested antioxidants, blood PAF levels can increase in inflammatory situations and during oxidative stress by peroxidation of phospholipid cellular membranes and LDL oxidation [10]. Notably, it has been demonstrated that endogenous or ingested PAF inhibitors can inhibit the actions of PAF [10,93]. However, the absence of circulating antagonists may result in increased PAF activity [5]. Research relating to PAF antagonists will be further explored in Section 4.

PAF and PAFLL can induce the release of reactive oxygen species (ROS) [10,94,95] that can lead to LDL oxidation [96]. Likewise, LDL oxidation is responsible for an increase in PAF levels [97] and oxidised LDL contains PAFLL [98]. This is significant as low concentrations of intact oxidised LDL have the ability to activate platelets through a mechanism mediated by PAFLL and the PAF-R [99]. Juxtaposed, it seems that PAF-AH in high-density lipoproteins (HDL) protects against the activity and production of oxidised LDL by hydrolysing PAF and PAFLL, thus reducing atherogenic changes in LDL and related inflammatory processes [10,100,101,102]. However, it was also demonstrated that upon LDL oxidation PAF-AH is gradually inactivated [97] potentially by oxygen radicals [103], thereby hindering its ability to suppress the proinflammatory activities of PAF and PAFLL [97]. It is clear that since Demopoulos’ et al. [10] hypothesised that PAF is the molecular link between atherosclerosis theories, there has been a series of reviews arguing that PAF is a central mediator in cardiovascular inflammation [5,11,104,105]. However, further research is still required to fully establish the role PAF and its related metabolic enzymes in atherosclerosis and CVD in order to develop effective preventative and therapeutic strategies.

**Figure 3:**
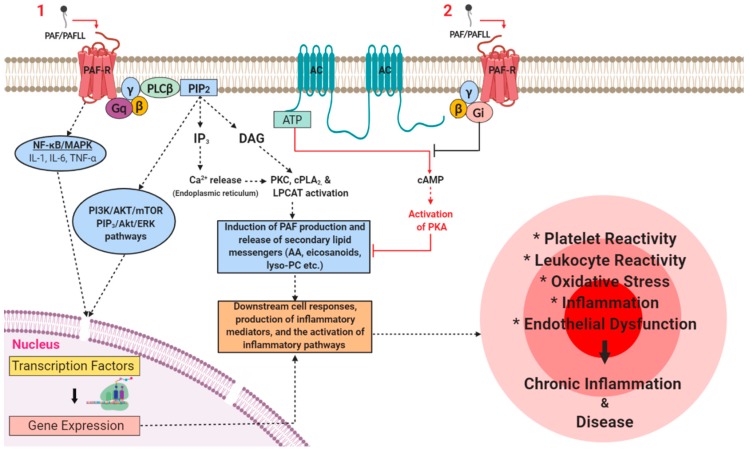
A simplified schematic that illustrates the main pro-inflammatory signalling pathways that PAF induces through binding with its receptor under certain stresses or stimuli in various pathways and inflammatory cascades in inflammation-related chronic disorders.

Systemic inflammation is also the underlying cause and driving process accountable for several other chronic diseases including cancer of which PAF and its receptor plays a significant role [8,106]. Apart from inducing inflammatory signalling, PAF also plays a significant role in suppressing the immune system, promoting metastasis, and supporting tumour growth by altering local cytokine and angiogenic networks [8,107,108]. Indeed, PAF is responsible for the activation of the NF-κB pathways, PAF overexpression in various tumours, and promotion of inflammation and angiogenesis in the tumour microenvironment [8,108,109,110]. There are several studies indicating that PAF and its receptor are critical components of the molecular processes in the development and progression of breast cancer [111,112], colorectal cancer [113], oesophageal cancer [58], lung cancer [59], liver cancer [60], pancreatic cancers [114], skin cancers [115], and various other cancers [8,107]. This has led to significant interest among researchers to develop novel therapeutics relating to PAF and its metabolism [8,116]. As aforementioned, PAF seems to be critical in cell signalling in the CNS. Therefore, it is unsurprising that PAF is also involved in the development of neurological and neurodegenerative disorders such as Alzheimer’s disease and potentially Parkinson’s diseases [117]. Indeed, PAF is also a well-established mediator in a plethora of other inflammation-related diseases including allergies [118,119] and anaphylaxis [12,19,120], HIV [55,121], sepsis [12,122], chronic obstructive pulmonary disease (COPD) [123,124], bacterial [125,126,127] and viral infections [128], lung pathology relating to smoking [123], asthma [129,130], periodontitis [131], and renal disorders [132]. A simplified example of PAF-induced pro-inflammatory cell signalling is outlined in Figure 3.

The PAF-R is expressed on the membranes of several cell types that are central to physiological and pathophysiological responses such as platelets, endothelial cells, monocytes, and macrophages. Several risk factors can increase the synthesis of PAF and PAFLL and upregulate the expression of the PAF-R. Activation of PAF-R signalling through Gq-linked mechanisms initiates PLCβ-mediated hydrolysis of PIP_2_ leading to the formation of DAG and IP_3_ and subsequently to a transient increase of CA^2+^ released from intracellular stores and the activation of PKC. The rise in Ca^2+^ activates cPLA_2α_ that leads to the release of lysophosphatides and AA that can be used as a substrate for the further synthesis of PAF and eicosanoids respectively. The activation of cPLA_2_ and the PAF biosynthetic enzymes (LPCAT) leads to additional synthesis of PAF and secondary lipid messengers. This results in the occurrence of a PAF cycle that further amplifies the initial inflammatory response and leads to the expression of pro-inflammatory genes that gives rise to the synthesis and release of various lipid mediators, cytokines, growth factors, ROS, reactive nitrogenous species (RNS), and the expression of integrins and selectins in the membranes of activated cells at the site of inflammation. Therefore, a rise in the levels of downstream mediators, PAF, and the subsequent further activation of the PAF/PAF-R pathways promotes the activation and aggregation of platelets and leukocytes, the activation of endothelial cells, increased leukocyte adherence, motility, chemotaxis, invasion, and migration. These processes culminate in the development of endothelial dysfunction, thus stimulating the onset and development of inflammation-related chronic diseases and disorders. Juxtaposed, PAF-R induced signalling through Gi-linked mechanisms inhibits the conversion of ATP to cAMP via adenylyl cyclase, thus preventing the activation of PKA and related anti-inflammatory signalling processes. Adapted with permission [5]. Abbreviations: AA, arachidonic acid; AC, adenylyl cyclase; AKT, protein kinase B; ATP, adenosine triphosphate; Ca, calcium; cAMP, cyclic adenosine monophosphate; cPLA_2_, cytosolic phospholipase A_2_; DAG, diacylglycerol; ERK, extracellular signal-regulated kinases; IL, interleukin; LPCAT, lysophosphatidylcholine acyltransferase; Lyso-PC, lyso-phosphatidylcholine; NF-κB, nuclear factor kappa-light-chain-enhancer of activated B cells; MAPK, mitogen-activated protein kinase; PAF, platelet-activating factor; PAFLL, PAF-like lipids; PAF-R, PAF-receptor; PI3K, phosphatidylinositol 3-kinase; PIP_2_, phosphatidylinositol 4,5-bisphosphate; PKA, protein kinase A; PKC, protein kinase C; PLCβ, phospholipase C-β; mTOR, mechanistic target of rapamycin; RNS, reactive nitrogenous species; ROS, reactive oxygen species; TNF-α, tumour necrosis factor-α.

## 4. The Potential Use of Platelet-Activating Factor Inhibitors as Therapeutics and Preventatives of Disease

Research into potential physiological and therapeutic ways of suppressing PAF activity demonstrated that endogenous or ingested PAF inhibitors could inhibit the actions of PAF [10,93]. Endogenous inhibitors of PAF have been identified in humans [133], many of which were identified as cardiolipins [134,135]. As a consequence of discovering that the body circulated PAF antagonists, it was thought that the absence of circulating antagonists could result in increased PAF activity [5]. Therefore, the potential role of PAF inhibitors in disease prevention and treatment has been of significant interest over the last three decades. Initial indications in the early 1980s demonstrated that PAF release from leukocytes could be modulated pharmacologically [136]. This was followed by studies using pharmacological compounds such as ticlopidine and calmodulin to study PAF-induced platelet aggregation [137,138]. At that time it was also shown that methanolic extracts of garlic bulbs exhibited inhibition of various platelet agonists including PAF [139]. This seems to be the first time in the literature that compounds originating from food were reported to have inhibited PAF-induced platelet aggregation. This was a significant finding as it demonstrated the existence of not only pharmacological therapeutics, but potentially dietary sources of PAF inhibitors also.

Around this period of PAF research there was a large increase in the number of published research relating to the discovery of PAF antagonists of natural and synthetic origin for which we now know of several hundred natural and synthetic PAF inhibitor molecules in existence [14]. In particular, researchers were investigating the potential use of compounds known as ginkgolides isolated from the *Ginkgo biloba* tree; a tree native to China, the existence of which dates back over 270 million years [140].

There are several ways to classify PAF inhibitors including if they are of natural of synthetic origin, they can be classified by their various chemical structures, and they can be classified by their interaction with the PAF-R, e.g., specific and non-specific inhibitors [141]. In terms of their structures, PAF inhibitors can be PAF analogues such as polar lipids, or there are molecules that are dihydropyridines, nitrogen heterocyclic compounds, phenolics, and other various natural medicinal compounds [141,142,143].

Along with being classified into compounds of natural or synthetic origin, PAF inhibitors can be characterised into two main classes according to their specificity: non-specific and specific inhibitors. Non-specific PAF inhibitors are compounds that inhibit certain processes in the PAF-induced signal transduction pathways such as calcium channel blockers, G-protein inhibitors, intracellular calcium chelators, etc. [14]. Various non-specific PAF inhibitors were crucial to identifying the individual steps of PAF-related signal transduction pathways. However, their pharmacological value is limited due to their low specificity [144,145,146,147]. By contrast, specific PAF inhibitors competitively or noncompetitively bind with the PAF-R. These types of inhibitors may have potential therapeutic value [5,14]. In Section 4.1 and Section 4.2 some of the most important natural and synthetic inhibitors and their specificity are discussed.

### 4.1. PAF Inhibitors of Synthetic Origin

The initial synthetic PAF inhibitor compounds such as CV-3988 [148,149], CV-6209 [150], RO 19-3704 [151], and ONO-6240 [152] were structurally similar to PAF. In fact CV-3988 a thiazolium derivative was a zwitterionic species that was the first synthetic antagonist of the PAF-R [148]. Later inhibitors replaced the glycerol backbone with cyclic structures such as SRI 63-441 [153], SRI 63-073 [154], UR-11353 [155], and CL-184,005 [156]. Subsequently, other PAF antagonists were developed that had no structural similarity to PAF. These antagonists were composed of heterocyclic structures that were characterised by sp^2^ nitrogen atom that interacted with the PAF-R as a hydrogen bond acceptor [141]. Many of these were derivatives of imidazolyl that lead to the development of lexipafant [157] and modipafant [158], thiazolidine derivatives such as SM-10661 [159], pyrrolothiazole-related antagonists such as tulopafant [160], and hetrazepine derivatives like WEB-2086 and WEB-2170 [161]. There are a plethora of synthetic PAF-R antagonists including psychotropic triazolobenzodiazepines [162], L-652,731 [163], and various examples of inorganic metal complexes [143,164]. However, it was later discovered that some of these antagonists were not orally active and some had toxicity issues [165,166], thus they had limited therapeutic value [167].

Clinical trials were conducted for several of these inhibitors, which demonstrated their tolerability and safety, but there were issues with their efficacy; juxtaposed, there were several trials that indicated positive outcomes following PAF-R antagonism. The inhibitors and their target diseases or disorders are outlined in Table 2.

Notably, some molecules exhibit dual antagonistic properties towards PAF and other inflammatory mediators. For instance, rupatadine is both an antagonist of the PAF-R and the histamine H(1) receptor [187], whereas LDP-392 can target both PAF and 5-lipoxygenase [188]. Likewise, common statins targeting CVD [189,190] and antiretrovirals targeting human immunodeficiency virus (HIV) [191,192] also exhibit anti-PAF pleiotropic effects. Indeed, various other molecules can inhibit both PAF and inducible nitric oxide synthase induction (iNOS) [193] or thromboxane synthases [194].

Finally, apart from the various compounds presented in Table 2, research has investigated the use of various inorganic metal complexes including other structurally related and structurally dissimilar PAF-R antagonists [141]. The authors recommend the following comprehensive reviews for further information on various synthetic and inorganic metal complexes with PAF-R antagonistic properties, their structures, synthesis, and biological effects [116,141,167,185].

### 4.2. PAF Inhibitors of Natural Origin

Extracts from *Ginkgo biloba* were some of the first PAF inhibitors of natural origin to be discovered. Several studies by Pierre Braquet and colleagues demonstrated that one compound in particular, BN 2021, was a highly specific competitive PAF antagonist. Several related ginkgolides also exhibited inhibitory properties against PAF [195,196,197,198,199,200]. Indeed, several other researchers at the time discovered anti-PAF properties in other natural isolates of Chinese medicinal herbs such as phomactin A, kadsurenone, and various xanthones [201,202,203,204,205]. In fact, the discovery that compounds from garlic bulbs possess anti-PAF activity stimulated interest in the exploration of natural compounds for anti-PAF activity [139].

By 1996, several molecules had been discovered with PAF-like activity as reviewed by Demopoulos [48]. Further experimentation uncovered that a neutral glycerylether lipid without an acetyl group from pine pollen exhibited biological activity against PAF [206]. Consequently, it was deduced that other lipid extracts could potentially inhibit PAF-induced platelet aggregation. This led to a series of studies investigating food lipid extracts starting around 1993, which initially lead to the discovery of PAF antagonists in the polar lipid fractions of olive oil [207], honey and wax [208], milk and yoghurt [209], mackerel (*Scomber scombrus*) [210], and wine [211] before the turn of the century. These studies deduced that mainly polar lipids such as glycerophospholipids and glycolipids exhibited potent inhibition against PAF-induced platelet aggregation through competitive binding to the PAF-R. As this research field developed it was noted that many of the compounds discovered that exhibited anti-PAF activity were also constituents of foods of the Mediterranean diet [5,212,213]. Therefore, these constituents may be responsible for the observed beneficial effects of consuming the Mediterranean diet [5,212,213]. Indeed, later research demonstrated that polar lipid extracts of olive oil, olive pomace, and fish could also affect many of the PAF metabolic enzymes both in vitro and in vivo [54,214,215]. These extracts were able to aid in the re-equilibration of PAF levels with beneficial outcomes against models of chronic inflammation.

Research into the effect of lipids on PAF activity and PAF metabolism is still being explored today in the pursuit of finding natural ways to prevent the pro-inflammatory signalling of PAF. It is now known that many foods, beverages, and other natural sources including food industry by-products are rich in PAF antagonists [142,216]. However, there have been several critical discoveries in vivo that suggest that PAF inhibitors of natural origin may help prevent diseases such as CVD. In studies in vivo, olive oil, olive oil polar lipids extracts, and olive oil neutral lipids extracts were administered to rabbits consuming an atherogenic diet. It was demonstrated that rabbits consuming olive oil or olive oil polar lipid extracts had more beneficial physiological and biochemical changes as a result of increased plasma levels of PAF-AH, less oxidation in the plasma, a reduction of atherosclerotic lesion thickness, and retention of vessel wall elasticity, thus impeding atherosclerosis development [217]. These results were corroborated in a subsequent study that found that polar lipid extracts of olive oil and olive pomace can impede early atherosclerosis development through reducing platelet sensitivity to PAF and reducing atherosclerotic lesion thickness [218]. A later follow-up study in rabbits demonstrated that olive pomace polar lipid extracts were equipotent to simvastatin in preventing the progression of atherogenesis [219].

**Figure 4:**
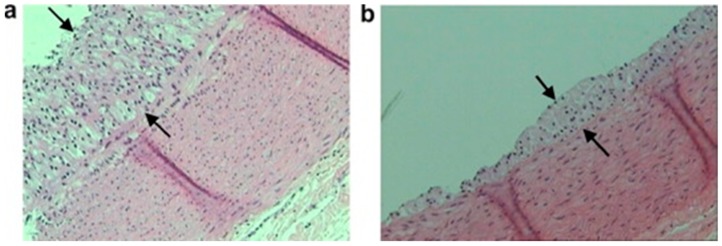
Representative optic micrographs ×100 of aortic wall sections stained with hematoxylin and eosin obtained from the two rabbit experimental groups. Atherosclerotic lesions appear as foam cells between the arrows. Each tissue sample was approximately 5 µm thick. (**a**) Group A (atherogenic diet) and (**b**) group B (atherogenic diet enriched with seabream polar lipids). Reproduced with permission from Nasopoulou et al. [220].

It was questioned whether other polar lipid extracts of natural origin could exhibit the same effects. Therefore, two studies of similar design demonstrated anti-atherogenic effects when rabbits consumed polar lipids extracted from fish (seabream, *Sparus aurata*) in a model of hypercholesterolaemia. These studies demonstrated that fish polar lipids could also reduce platelet aggregation, reduce atherosclerotic lesion size, and increase HDL levels in rabbits [220] along with modulating PAF metabolism leading to lower PAF levels and activity in rabbit blood [215]. Representative optic micrographs (×100) of the aortic wall of these rabbits are presented in Figure 4. These images demonstrate that rabbits consuming an atherogenic diet supplemented with fish polar lipids leads to a reduction of atherosclerotic lesion width (b) versus a control group that consumed only an atherogenic diet (a) [220].

However, after discovering that polar lipids could inhibit PAF in vitro and in vivo, the question remained whether these compounds of natural origin could affect human health? It is now known that there have been some promising nutritional trials that indicate that PAF antagonists in wine may affect platelet aggregation and metabolism postprandially in humans [43,221]. In people with metabolic syndrome, consumption of meals including wild plants of the Mediterranean diet rich in PAF inhibitors postprandially reduced PAF-induced platelet aggregation [222]. Other results from dietary intervention studies have shown that the administration of traditional Mediterranean diet meals [223,224] to either normal volunteers or individual’s with type II diabetes mellitus (who have a predisposition to CVD) resulted in the characteristic lower PAF activity in blood (measured as PAF-induced platelet aggregability), which correlates with inhibition of atherogenesis according to experiments [217].

Likewise, dietary supplements can reduce PAF-induced platelet aggregation and increase PAF catabolism in healthy humans [225]. These studies collectively indicate that consumption of PAF antagonists from foods and nutraceuticals may benefit the consumer by reducing the pro-inflammatory effects of PAF either through inhibition of PAF/PAF-R signalling or by influencing the metabolic enzymes of PAF.

Considering, the potential use of dietary polar lipids for the prevention of CVD, several recent studies have discovered PAF antagonists in various fish species and by-products of the fishing industry including salmon fillet and head, minced boarfish, and herring [226,227,228], and other foods such as sheep and goat meat [229], milk and fermented dairy products [230,231,232,233], and beer and brewing by-products [234,235]. Future research in this area aims to develop novel functional foods and nutraceuticals that incorporate these bioactive polar lipid extracts for the prevention of CVD and other inflammation-related diseases. For more extensive reviews of the anti-inflammatory and antithrombotic properties of various food polar lipids the authors suggest the following literature [49,142].

## 5. Current Trends in Platelet-Activating Factor Research

The latest published research in relation to PAF is vast and traverses a plethora of biological systems and pathways. The following sections provide a brief overview of some of the most exciting PAF research. However, the topics discussed are not exclusive and an effort has been made to include various related fields of research.

### 5.1. A Potential Anti-Inflammatory Role of PAF

While the majority of the studies relating to PAF research focus on the pathological consequences of the PAF/PAF-R pathways, there is some evidence that PAF also has anti-inflammatory effects [8]. A recent study demonstrated that TNF-α promotes intestinal mucosal repair by upregulating the PAF-R in the intestinal epithelium [7]. Similarly, in some cancers PAF exhibits beneficial effects. Elevated expression of the PAF-R enhances apoptosis via activation of the NF-κB pathways [236,237] and through the dual action of the NF-κB pathway in malignancy and apoptosis via the immune response [106,238]. Loss of the PAF-R in mice beneficially augmented PMA-induced inflammation and chemically induced carcinogenesis, which seems to indicate that the PAF-R suppresses inflammation and neoplastic development in response to chemically induced carcinogenesis [239]. It is imperative for the development of future therapeutics that these potential anti-inflammatory properties of the PAF/PAF-R pathways are more intensively investigated.

### 5.2. PAF and Cancer

Research regarding the role of PAF in cancer has led to several interesting discoveries over the last twenty years. PAF is a critical mediator of many cancers [8]. However, it is becoming clear that PAF plays a significant role in cancers that are particularly difficult to treat. Melanoma for instance is characterised as the most dangerous form of skin cancer due to its capacity to rapidly metastasise as a result of pro-inflammatory signalling that is mediated by PAF/PAF-R [109,240,241]. What has recently become apparent is that pro-oxidative stressors can suppress host immunity through their ability to generate oxidised lipids and PAF-R agonists [8,242]. It has been demonstrated that PAF and PAFLL are generated by skin cells on exposure to UV light, thus contributing to the pathology of melanoma [243]. Indeed, it seems cruelly ironic that PAF and PAFLL are also generated by tumour cells in melanoma patients following exposure to radiation treatment [242]. Structural analysis of these PAF-R agonists revealed that radiation therapy leads to the nonenzymatic production of multiple oxidised glycerophosphocholines (PAFLL) and PAF itself [242].

Other studies have found similar findings [9], whereby PAFLL are generated by radiotherapy, and that their action on tumour cells protects them from radiation induced cell death by affecting macrophages. Such PAFLL molecules stimulate tumour growth through immunosuppression [9]. Therefore, the association of radiotherapy with the PAF-R antagonists represents a promising strategy for improving the efficacy of radiotherapy [8,9]. Additionally, it has been observed that there is elevated expression of the PAF-R in cervical cancer patients post-surgery [244]. In the same study it was reported that higher levels of PAF-R mRNA and protein were expressed by squamous carcinoma cell lines and cervical cancer-derived cell lines than immortalised keratinocytes. Gamma radiation increased PAF-R expression and the generation of prostaglandin E_2_ and PAF-R ligands in these tumour cells. Inhibition of PAF-R signalling by CV-3938 prior to irradiation led to the inhibition of prostaglandin E_2_ and an increase of tumour cell death. Furthermore, human carcinoma cells transfected with PAF-R were more resistant to radiation compared to cells lacking the PAF-R. PAF antagonist CV-3988 inhibited the production of prostaglandin E_2_ in irradiated cells transfected with PAF-R. As a consequence, it was deduced that irradiation of carcinoma cells leads to the synthesis of PAF-R ligands and higher expression of the PAF-R that protects tumour cells from death, and suggests that a combination of radiotherapy with PAF-R antagonists could be a promising target for cancer treatment [244]. There are several studies indicating that PAF-R antagonists could also potentially be used as an adjuvant treatment to chemotherapy in cancer [115,245,246] or to treat common side effects of chemotherapy [247]. A new approach in this field is the use of PAF inhibitors such as metal compounds, which may have additional direct anticancer properties (a combined anti-PAF and anticancer activity), to amplify the effectiveness of common anticancer treatments [8,141,143].

### 5.3. Current Research Trends on PAF and PAFLL in Cardiovascular Disease

One of the most topical debates in PAF-related research currently is the role of PAF metabolism in CVD. PAF biosynthesis, and transport is a tightly regulated process by which enzymatic reactions involving intracellular and extracellular PAF-AHs terminate signals in the PAF signalling cascade by selectively degrading PAF and PAFLL [3,12]. There is considerable debate about PAF-AH or the plasma form lipoprotein-associated phospholipase A_2_ (Lp-PLA_2_) and their role in in the development of atherosclerosis [65]. PAF-AH is a catabolic enzyme of PAF and thus has an anti-inflammatory function. However, HDL-associated PAF-AHs is thought to be anti-inflammatory and antiatherogenic by its reduction of monocyte adhesion to the endothelium, its capacity to attenuate phospholipid oxidation, and its ability to impede the biological activity of minimally modified LDL [105]. On the other hand, LDL-associated PAF-AH is considered pro-inflammatory and its role in atherosclerosis is controversial.

Functionally, PAF-AHs hydrolyse glycerophospholipids at the *sn*-2 position, with no preference for the type of linkage present at the *sn*-1 position (acyl or alkyl). Hydrolysis of glycerophospholipids by PAF-AH generates lyso-PAF or lysophosphatidylcholine (lyso-PC) and short or oxidised fatty acids, many of which tend to exhibit pro-inflammatory properties [248]. This led some researchers to consider that PAF-AH may contribute to vascular inflammation due to the generation of these pro-inflammatory molecules [64,65]. As a result, there was a realisation that inhibition of PAF-AH could prevent vascular inflammation [249]. This in turn led to the development of several PAF-AH inhibitors, including darapladib that has been tested in clinical trials as an adjunct treatment to cholesterol-lowering therapies for its capacity to stabilise atherogenic plaques [250,251,252]. These studies and others in vitro, in vivo, and in humans have had varying success and it is postulated that darapladib may exert pleiotropic effects, these findings and more have been extensively reviewed [64,65,248]. It is also hypothesised that another future strategy to inhibit PAF-AH could involve RNA interference (RNAi), which was shown to ameliorate atherosclerosis in apolipoprotein E-deficient mice [253,254]. All things considered the debate surrounding the role of PAF-AH in CVD continues to develop.

On another topical issue, over the past two decades there has been significant breakthroughs in the understanding of the role of oxidised phospholipids (oxPL) and PAFLL in CVD. Lipid oxidation products such as oxPL, many of which resemble PAFLL, play a role in various normal and pathological states [255]. The oxPL acquire different biological activities uncharacteristic of their unoxidised precursors [256]. For instance, oxPL play a role in angiogenesis, endothelial barrier function, regulation of innate and adaptive immunity, and thrombosis [255,257]. However, oxPL are more well-known for their role as inducers of systemic inflammation and atherosclerosis [105,258]. Indeed, bioactive lipids including PAF, PAFLL, oxPL, and lyso-PC are even present in atherosclerotic plaque [259]. PAF-AH degrades pro-inflammatory oxPL and plays a key role in the generation of lyso-PC and oxidised fatty acids [105]. In addition to their pro-inflammatory actions, oxPL can promote anti-inflammatory and tissue-protective mechanisms, depending on the biological situation [258,260].

OxPL carry out their functions by binding to pattern-recognition receptors (PRR) that are found on the cell surface [261]. These include toll-like receptors (TLR) and scavenger receptors. OxPL can also circulate in the blood stream interacting with C-reactive protein (CRP) [262], lipopolysaccharide binding protein (LPB), or plasma CD14 [260]. Some oxPL can interact with the PAF-R and induce platelet activation [263]. Current research trends in this field are still interested in discerning the pro-inflammatory nature of oxPL. However, there is also significant interest in the anti-inflammatory actions of oxPL, which function by inhibiting inflammatory signalling pathways via NRF2-dependent and -independent mechanisms, upregulation of genes associated with endogenous antioxidants, antagonism of TLR and a host of other mechanisms as reviewed by Mauerhofer et al. [264]. In addition, PAF and PAFLL carry out their functions by binding to TLR, who’s signalling is associated with the signalling of new PAF production. On the other hand, the PAF receptor appears to also mediate signalling in the pathogenesis of inflammatory diseases through other molecules outside of PAF and PAFLL such as lipoteichoic acid (LTA) and various lipopolysaccharides (LPS). This is reviewed by Detopoulou et al. [213] who discuss the role of PAF and TLR in the crosstalk of dyslipidaemia, inflammation, and atherogenesis.

Other research trends are focused on detecting and quantifying the levels of oxPL and PAFLL in models of disease, the plasma of diseased patients or patients receiving treatments. For instance, the mass spectrometry analysis of plasma oxPL in diabetes patients [265], the level of plasma oxPL in Alzheimer’s disease patients treated with a carotenoid supplement [266], or PAFLL in coronary artery disease patients at risk of cognitive decline due to depression [267]. However, one of the current major challenges of oxPL, PAF, and PAFLL research is the development of simplified mass spectrometric procedures for high-throughput and affordable analysis [268,269]. Certainly, there are several limitations to PAF quantification including sensitivity, pre-analysis derivatization, interference with isobaric molecules, and the fact that it is expensive to conduct [269]. Therefore, further research is required to develop reliable, inexpensive, and reproducible methods to further advance this research field.

### 5.4. Current Research Trends on PAF in Neurological Disorders

PAF also seems to be involved in the development of CNS disorders. The latest research indicates that PAF alters blood–brain barrier permeability, which may have implications for CNS inflammatory disorders [270]. Indeed, PAF may be implicated in the development of cerebral dysfunction following traumatic brain injuries [271] and PAF-R mediated signalling may affect postsynaptic hippocampal injury in encephalomyelitis [272]. PAF also seems to be involved in neurodegenerative diseases such as amyotrophic lateral sclerosis [273]. There is evidence that PAF is critical to inflammatory signalling in pain, spinal cord injury, and traumatic brain injury itself [274,275,276]. Finally, as PAF is a mediator of various neurodegenerative diseases it is imperative to understand PAF-mediated cell signalling and its inhibition. For instance, in the early stages of Alzheimer’s disease, cognitive decline and synapse loss seem to be inhibited by ginkgolides A and B, which are PAF-R antagonists [277]. Likewise, several studies have suggested that PAF-R antagonists may be beneficial in the treatment of Parkinson’s disease and prion-induced synapse degradation [117,278,279].

In relation to neurological issues, early studies indicated that injecting PAF into peripheral tissues such as skin enhanced pain sensitivity in animals and humans [276,280]. Notably, there are now several studies implicating PAF in pain signalling due to the role of PAF in regulating various functions of cells in the peripheral tissues and the CNS [276]. As such, the PAF/PAF-R signalling cascade seems to be involved in tissue injury-induced pain and neuropathic pain [276]. Indeed, there is some evidence to suggest that PAF antagonists may be anti-allodynic [281]. Animals studies have also shown that PAF antagonists (TCV-309) alone or in combination with opioids reduced pain in animal models of bone cancer pain indicating that they may have palliative properties [282]. Recently it has been determined that lysophosphatidylcholine acyltransferase (LPCAT)2 along with PAF antagonists (ABT-491) may be a novel therapeutic target for analgesic drugs due to the fact that it’s deficiency in partial sciatic nerve ligation seemed to attenuate pain in mice [283].

### 5.5. Current Research Trends on PAF in Renal and Urinary System Disorders

The role of PAF in renal function and pathology dates back to when the structure of PAF was first being determined. Snyder and colleagues described a molecule they termed APRL, which was later identified as being PAF. In renal physiology, PAF is considered one of the main inflammatory mediators [284]. PAF is synthesised in the kidney by various renal cells including mesangial cells but is also present due to infiltrating inflammatory cells [54,285]. PAF does not accumulate in the renal cells, but it is secreted and affects mesangial cells, neighbouring podocytes, and other infiltrating cells by binding to the PAF-R and inducing its signalling pathways [5]. Excessive production of PAF can lead to damage of these cells, thus inducing glomerulosclerosis and proteinuria [132]. PAF may also play a role in renal haemodynamics [286]. PAF antagonists have exhibited promising results in renal disorders [132] and components of the Mediterranean diet seem to exhibit effects on PAF metabolism in relation to the renal system [54,214]. Notably, vitamin D and its analogue paricalcitol exhibited strong anti-PAF effects in human cells. Administration of paricalcitol in haemodialysis patients for one month reduced PAF synthesis and increased PAF catabolism, which was accompanied with a reduction of PAF levels, renal inflammation [56]. However, further research is required in this field to fully understand how PAF/PAF-R signalling affects renal pathophysiology to develop novel treatments for glomerulosclerosis and proteinuria.

Interestingly the kidneys are not the only organ in the renal system affected by PAF and its metabolism. In humans, it is thought that cigarette smoking leads to alterations in urethral cells that share similar histology to urethral cells of interstitial cystitis and bladder pain syndrome (IC/BPS) patients through an inflammatory pathway mediated by PAF [287]. Overall it is demonstrated that PAF signalling is upregulated in IC/BPS and that cigarette smoke exposure further upregulated this pathway [287]. Future research aims to determine the role of PAF in IC/BPS development, whether PAF can act as a marker of IC/BPS, and if PAF antagonists may have therapeutic value in IC/BPS [288]. Likewise, PAF seems to be implicated in the development of bladder cancer [287]. This is unsurprising as several lines of research indicate that smoking cigarettes seem to induce the generation of PAF and PAFLL in other cancer pathologies including breast cancer [289,290]. What is most significant about the series of studies relating to cigarette smoke is that it is clear that PAF activity and metabolism is directly affected by lifestyle choices. Worryingly, in relation to the latest trend of consuming E-cigarettes, PAF-R expression is increased following their use, which may increase one’s risk of pneumococcal infections [291]. This may have untold health consequences to E-cigarette users over time. On a positive note, current research interests seem to be trending towards determining the role of lifestyle factors such as the use of E-cigarettes and other environmental stressors on PAF activity and metabolism [46].

## 6. New Frontiers in PAF Research

Over the last two years there have been remarkable discoveries concerning the PAF/PAF-R relationship. Indeed, the PAF-R structure has been elucidated and there is evidence that PAF induced signal transduction independent of the PAF-R as elaborated further in Section 6.1 and Section 6.2. These discoveries will open the PAF research field to new possibilities and greater understanding of the pathophysiological roles and functions of PAF and its receptor.

### 6.1. PAF-R Strucutral Elucidation

One of the most significant recent achievements in PAF research has been the elucidation of the structure of the PAF-R. Previous research was unable to determine the structure of human PAF-R. Instead, bovine rhodopsin was used as a model of the PAF-R [164]. Cao, Tan, Zhang, Wu, and colleagues recently solved the crystal structures of human PAF-R in complex with a PAF antagonist SR 27,417 and a PAF inverse agonist ABT-491 [292]. This is extremely important for GPCR research as only seven other lipid receptor structures have been elucidated [293,294]. Gaining a greater understanding of the PAF-R structure and its capacity to bind various ligands will allow for the development of effective therapeutic strategies against the PAF/PAF-R pathways. Moreover, this research will pave the way for the elucidation of other GPCR structures that are critical in physiology and pathophysiology.

### 6.2. Induction of Inflammatory Pathways Independent of the PAF-R

Finally, the majority of the pro-inflammatory effects of PAF are due to PAF binding to the PAF-R. However, recently it has been discovered that PAF can mediate NLRP3 (nucleotide-binding oligomerization domain, leucine-rich repeat–containing receptor family pyrin domain-containing 3)-NEK7 (NIMA-related kinase 7) inflammasome induction independently of the PAF-R [295]. Notably, PAF and PAFLL can activate the inflammasome resulting in IL-18 and IL-1β maturation, which is dependent on NLRP3, ASC (apoptosis-associated speck like protein containing a caspase recruitment domain), caspase-1, potassium efflux, and calcium influx. These findings are significant as they may explain why despite promising data, PAF antagonists have previously failed to exhibit clinical benefit in clinical trials relating to PAF-mediated inflammation in sepsis, acute pancreatitis, and asthma [171,296,297,298]. Additionally, the PAF-R modulates colitis-induced pulmonary inflammation through the NLRP3 inflammasome [299]. Considering the link between PAF/PAF-R and activation of the NLRP3 inflammasome, further research is required to discern whether PAF also activates the NLRP3 inflammasome in atherosclerosis [300].

## 7. Conclusions and Future Research Perspectives

It is clear that the discovery and structural elucidation of PAF sparked major interest into the role of PAF in physiology and pathophysiology, along with lipid mediators in general from the 1970s onwards. When the role of PAF in various diseases became clear researchers began to search for and design molecules to inhibit the actions of PAF. The aim of this article was to try present an overall picture of some of the historical perspectives and current research trends in relation to PAF research. It is clear from the wealth of evidence presented that understanding the mechanisms of PAF/PAF-R signalling in health and disease has yet to be fully elucidated. However, the discovery of various PAF and PAFLL agonists and antagonists, the role of the PAF metabolic enzymes in diseases, solving the human PAF-R structure, and identifying PAF signalling mechanisms independent of the PAF-R are some of the many major achievements in current PAF research. Nevertheless, despite all of these achievements, clinical trials have failed to demonstrate the efficacy of PAF-R antagonists in the treatment of inflammatory diseases. Similarly, lipidomic research has yet to provide a reliable, reproducible, and inexpensive method of PAF and PAFLL identification and quantification to be used as a biomarker of inflammatory diseases. These are just some of the many challenges that exist in PAF research. The importance to research PAF and its related signalling processes lies in the fact that it is involved in so many inflammation-related diseases, particularly CVD. However, as per Section 6, there have been critical breakthroughs in PAF research that hold significant promise in this research field. Future PAF research will most likely target PAF in conjunction with various other inflammatory mediators for novel multi-modal therapeutics. Therefore, it is clear that there is an abundance of research yet to be conducted to fully understand the mechanisms induced and governed by PAF and its metabolism in physiology and pathophysiology.

## Figures and Tables

**Table 1:** International conferences of platelet-activating factor (PAF).

Title	Date	Location
1st International Symposium on Platelet-Activating Factor and Structurally Related Ether-Lipids	26–29 June 1983	Paris, France
2nd International Conference on Platelet-Activating Factor and Structurally Related Ether-Lipids	26–29 October 1986	Gatlinburg, Tennessee, USA
3rd International Conference on Platelet-Activating Factor and Structurally Related Ether-Lipids	8–12 May 1989	Tokyo, Japan
4th International Congress on Platelet-Activating Factor and Related Lipid Mediators	22–25 September 1992	Snowbird, Utah, USA
5th International Congress on Platelet-Activating Factor and Related Lipid Mediators	12–16 September 1995	Berlin, Germany
6th International Congress on Platelet-Activating Factor and Related Lipid Mediators	21–24 September 1998	New Orleans, Louisiana, USA
7th International Congress on Platelet-Activating Factor and Related Lipid Mediators	24–27 September 2001	Tokyo, Japan
8th International Congress on Platelet-Activating Factor and Related Lipid Mediators	6–9 October 2004	Berlin, Germany
6th International Conference on Phospholipase A_2_ and Lipid Mediators	10–12 February 2015	Tokyo, Japan

**Table 2:** A list of some of the major synthetic PAF antagonists assessed against several conditions in clinical trials.

PAF-R Antagonist	Target Disease or Disorder	Outcome	Reference
Lexipafant	Cognitive impairment complications as a result of coronary artery bypass graft	No significant reduction in cognitive impairment	[168]
	Myocardial infarction	No significant effect on streptokinase-induced hypotension in myocardial infarction patients	[169]
	Sepsis	No significant affect in patients with severe sepsis	[170]
	Organ failure related to pancreatitis	No significant amelioration of systemic inflammatory response syndrome in pancreatitis-induced organ failure	[171]
Modipafant	Asthma	No significant effect against chronic asthma	[158]
	Asthma	No significant effect in early or late responses to allergens	[172]
	Responses to inhaled PAF	Potent inhibition of airway and neutrophil responses to PAF with a duration of up to 24 h and a reduction of secondary eicosanoid production in response to inhaled PAF	[173]
SR27417A SR27417A	Asthma	Modest inhibitory effects against asthma	[174,175],
	Ulcerative colitis	No evidence of efficacy in the treatment of acute ulcerative colitis	[176]
WEB 2086	Asthma	No attenuation of early of late allergen-induced responses or airway hyperresponsiveness	[177]
	UVB-induced dermatitis	Significant inhibition of UVB light-induced erythema	[178]
BN 50730	Rheumatoid arthritis	Ineffective in the treatment of rheumatoid arthritis	[179]
BN 52021	Pulmonary function in the early post ischaemic graft function in clinical lung transplantation	Improvement of alveoloarterial oxygen difference and a reduction of PAF levels	[180]
Ro 24-238	Psoriasis	No significant effects reported	[181]
TCV-309	Septic shock	No significant difference in adverse events or mortality. A substantial reduction of organ dysfunction and morbidity associated with septic shock was reported	[182]
Levocetirizine	Chronic idiopathic urticaria	Reduction of urticarial activity score	[183]
Rupatadine	Chronic idiopathic urticaria	Reduction of urticarial activity score but not as effective as levocetirizine	[183,184]
	Allergic rhinitis and allergies	Significant effects against both conditions as demonstrated in the comprehensive review by Mullol et al.	[185]
Y-24180	Asthma	Improvement of bronchial hyperresponsiveness in patients with asthma	[186]
